# Synthesis of pyrrolo[3,2-*a*]phenazines from 5-nitroindoles and anilines

**DOI:** 10.1007/s00706-013-1087-3

**Published:** 2013-10-10

**Authors:** Zbigniew Wróbel, Michał Więcław, Robert Bujok, Krzysztof Wojciechowski

**Affiliations:** 1 Institute of Organic Chemistry, Polish Academy of Sciences, ul. Kasprzaka 44/52, P.O. Box 58, 01-224 Warsaw, Poland; 2 Department of Chemistry, Warsaw University of Technology, ul. Noakowskiego 3, 00-664 Warsaw, Poland

**Keywords:** Amines, Anions, Heterocycles, Cyclizations, Nucleophilic substitutions, Lewis acids

## Abstract

**Abstract:**

Anilines react with 5-nitroindoles in the presence of *t*-BuOK in* N*,*N*-dimethylformamide (DMF) to form 5-nitroso-4-arylaminoindoles that in turn when treated with *N*,*O*-bis(trimethylsilyl)acetamide cyclize to pyrrolo[3,2-*a*]phenazines. In an alternative approach pyrrolo[3,2-*a*]phenazines are formed from aminoindoles and nitroarenes.

**Graphical abstract:**

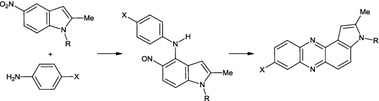

## Introduction

Phenazine derivatives are an important class of condensed heterocycles of natural origin [1–4]. Selected methods of synthesizing the phenazine framework are presented in Scheme 1. One of the oldest methods is the reaction of anilines with nitroarenes under basic conditions (the Wohl–Aue reaction, path a) [5]. The Holliman synthesis of phenazines (path b) is a base-induced cyclization of *ortho*-nitrodiphenylamines [6]. In the Bamberger–Ham reaction (path c) nitrosobenzenes dimerize under acidic conditions to form phenazines [7]. Other methods are the condensation of *ortho*-phenylenediamines with *ortho*-quinones (path d) [8], reaction of benzofuroxanes and phenols (the Beirut reaction, path e) [9], and palladium-catalyzed cyclization of 2-amino-2′-bromophenylenediamines (path f) [10].

**Figure:**
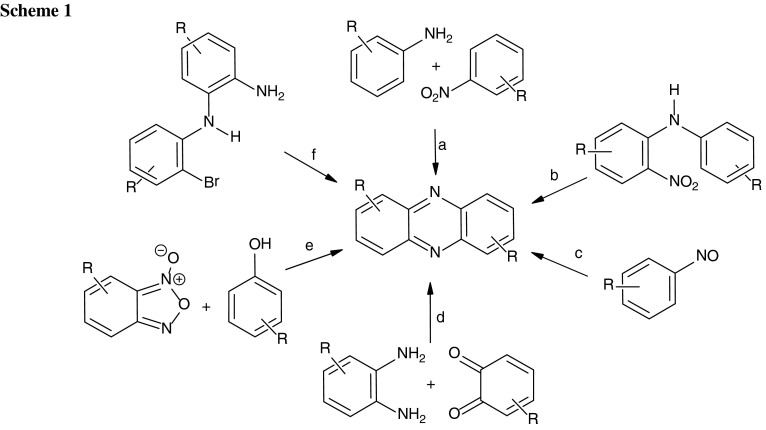

The classic Wohl-Aue synthesis of phenazines consists in the reaction of anilines with nitroarenes under harsh basic conditions, usually by heating of both starting materials with sodium or potassium hydroxide at 200 °C [5]. In recent years we extensively studied nucleophilic aromatic substitution reactions of hydrogen in nitroarenes [11–15]. During these studies we have found that anilines react with nitrobenzene derivatives under mild conditions in the presence of *t*-BuOK in DMF at −50 °C to form 2-nitrosodiphenylamines that in turn upon treatment with acetic acid cyclized to phenazines (Scheme2) [16, 17].

**Figure:**
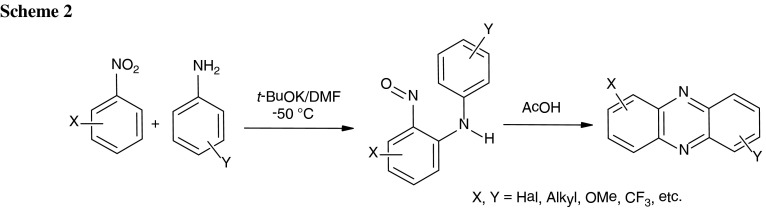

Other transformations of 2-nitrosodiphenylamines into heterocyclic systems developed by us include reactions with benzyl aryl sulfones to form 1,2-diarylbenzimidazoles [18] and cyclocondensation with functionalized alkyl acetates, such as malonates, phenyl- and phosphonyl-acetates, leading to 1-arylquinoxalin-2(1*H*)-ones [16, 19].

1,2-Benzo- and 1,2-heteroaryl-fused phenazines are of interest owing to their potential biological activity, as intercalators [20, 21], and antimicrobial agents [22, 23]. Reports on the synthesis of pyrrolo[3,2-*a*]phenazines are scarce. 1-(2-Aminoethyl)pyrrolo[3,2-*a*]phenazine was formed from 1,2-phenylenediamine and the 4,5-indoloquinone arising from electrochemical oxidation of 5-hydroxytryptamine [24]. Dipyrrolo[3,2-*a*:3,2-*h*]phenazines were synthesized in the oxidative dimerization of 5-aminoindoles [25]. Some pyrrolo[3,2-*a*]phenazine-10-carboxamides, obtained from 4-aminoindole and 2-iodo-3-nitrobenzoic acid, were tested as cytotoxic agents [26].

## Results and discussion

In this paper we present a simple synthesis of pyrrolo[3,2-*a*]phenazines from nitroindoles and anilines. Thus when we treated 5-nitroindole derivatives **1** and anilines **2** with *t*-BuOK in DMF at −50 °C, the expected 4-(*N*-arylamino)-5-nitrosoindoles **3** were formed in good yields (Scheme 3 and Table 1).

**Figure:**
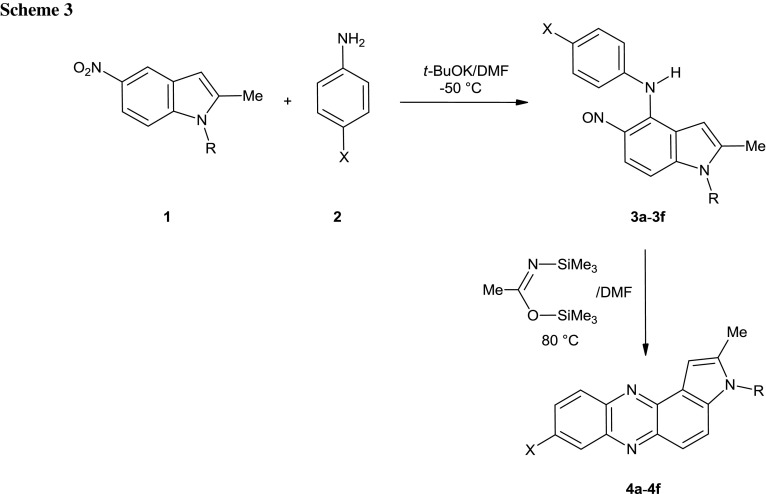

**Table 1:** Synthesis of nitrosoindoles **3** and pyrrolo[3,2-*a*]phenazines **4**

	R	X	Yield of **3**/%	Yield of **4**/%
**a**	Me	Cl	65	65
**b**	CH_2_Ph	Cl	36^a,b^	88
**c**	*n*-C_8_H_17_	CH_3_	30	88
**d**	*n*-C_8_H_17_	Cl	58	80
**e**	*n*-C_8_H_17_	OCH_3_	50	71
**f**	*n*-C_8_H_17_	CF_3_	–^b^	34

^a^Yield of the crude product

^b^The crude product without purification was subjected to cyclization to phenazine

Some of these compounds (**3b** and **3f**) proved unstable and thus after isolation without further purification they were used in the next step to form phenazines. The ^1^H and ^13^C NMR spectra of the obtained nitrosoamines **3** and **7** deserve some comments. In the spectra of some of these compounds we observed broadening of the signals corresponding to the protons and carbon atoms of the nitroso-substituted moiety and thus their full interpretation was troublesome. Such a signal broadening is probably due to a slow rotation of the nitroso group around the C–N bond. A similar phenomenon was observed in the NMR spectra of 2-(alkylamino)- and 2-(arylamino)nitrosobenzenes [27, 28].

In our earlier papers we have shown that cyclization of *N*-(2-nitrosophenyl)anilines to phenazines proceeds satisfactorily in boiling acetic acid [16, 17], with K_2_CO_3_ in methanol at room temperature [17], or with *N*,*O*-bis(trimethylsilyl)acetamide (BSA) [17]. Attempted cyclization of the model nitroso compound **3d** in boiling acetic acid was unsuccessful; the starting material was consumed within 90 min (TLC control) but no defined products were obtained. No reaction of **3d** was observed in the presence of K_2_CO_3_ in methanol. The cyclization of **3d** occurs satisfactorily in the presence of BSA in DMF at 80 °C giving the expected pyrrolophenazine **4d** in good yield. These reaction conditions were adapted to reactions of other 4-(*N*-arylamino)indoles **3**. The results are summarized in the Table 1.

Alternatively, the pyrrolo[2,3-*a*]phenazines can be obtained from aminoindoles and nitroarenes (Scheme 4). Thus, when we reacted 4-aminoindole **6a** with 4-nitroanisole (**5**) under standard conditions (*t*-BuOK/DMF, −50 °C) the expected nitrosoaniline **7a** was formed. Since the amine **7a** proved unstable, it was without purification subjected to reaction with BSA and cyclized to 9-methoxypyrrolo[3,2-*a*]phenazine **4** **g** that was isolated in 90 % yield. Similarly 5-aminoindole **6b** and 4-nitroanisole formed the relatively stable nitroso derivative **7b** that was isolated in 40 % yield. Treatment of the compound **7b** with BSA led to isomeric 8-methoxypyrrolo[3,2-*a*]phenazine **4** **h** in 64 % yield.

**Figure:**
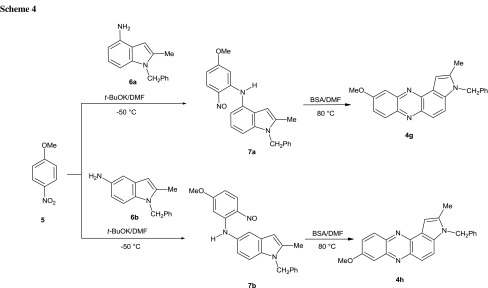

These reactions show the versatility of the proposed approach to pyrrolophenazines enabling the synthesis of derivatives bearing substituents in the desired position of the heterocyclic system, as exemplified by the synthesis of 8- and 9-methoxy derivatives **4** **g** and **4e** that can be obtained from different nitroarene–amine pairs, namely 5-nitroindole and *para*-anisidine or 5-aminoindole (**6b**) and 4-nitroanisole (**5**).

In summary, a novel two-step approach to pyrrolophenazines starting from easily available nitroindoles and anilines was developed. In an alternative reaction sequence the pyrrolophenazines can be obtained from nitroarenes and aminoindoles. The simplicity of this approach makes it an interesting alternative to other procedures.

## Experimental

All reactions were performed under argon atmosphere. ^1^H and ^13^C NMR spectra were recorded on Bruker 500 MHz spectrometer (500 MHz for ^1^H and 125 MHz for ^13^C spectra). Chemical shifts (*δ*) are expressed in ppm referred to TMS, coupling constants in Hertz. Mass spectra (EI, 70 eV) were obtained on an AMD-604 spectrometer. ESI mass spectra were obtained on SYNAPT G2-S HDMS. Merck silica gel 60 F_254_ plates were used for TLC. Merck silica gel 60 (230–400 mesh) was used for flash column chromatography.

### *Typical procedure for synthesis of compounds****3****and****7***

#### * N*-*(4*-*Chlorophenyl)*-*1,2*-*dimethyl*-*5*-*nitroso*-*1H*-*indol*-*4*-*amine* (**3a**, C_16_H_14_ClN_3_O)

4-Chloroaniline (0.32 g, 2.5 mmol) in 2 cm^3^ DMF was added to a solution of 0.67 g *t*-BuOK (6 mmol) in 10 cm^3^ DMF cooled to −50 °C. After 5 min a solution of 0.38 g 1,2-dimethyl-5-nitroindole (2 mmol) in 3 cm^3^ DMF was added. The reaction was stirred at −50 to −40 °C until the starting indole disappeared (1–2 h, TLC control, SiO_2_, toluene/ethyl acetate 10:1). Then the reaction mixture was poured into 100 cm^3^ water with 5 g NH_4_Cl. The precipitate was dissolved in 100 cm^3^ EtOAc and dried with Na_2_SO_4_. After evaporation of solvent the product was purified by column chromatography (SiO_2_, toluene/ethyl acetate). The product **3a** was obtained as a dark red solid; m.p.: >285 °C (decomp.); *R*_f_ = 0.18 (toluene/ethyl acetate 10:1); ^1^H NMR (500 MHz, CDCl_3_): *δ* = 2.21 (s, 3H), 3.61 (s, 3H), 5.41 (br s, 1H), 6.91 (br s, 1H), 7.15–7.26 (m, 2H), 7.37–7.38 (m, 2H), 8.14 (br s, 1H), 14.49 (s, 1H) ppm; ^13^C NMR (125 MHz, CDCl_3_): *δ* = 12.47, 30.00, 104.55, 105.57, 111.77, 127.35, 128.18, 128.99, 129.33, 132.57, 134.94, 137.38, 141.32, 153.62 ppm; MS (ESI): *m*/*z* = 300 ([M + H]^+^, 100), 282 (8); HRMS (ESI): calcd. for C_16_H_15_^35^ClN_3_O 300.0904, found 300.0905.

#### *1*-*Benzyl*-*N*-*(4*-*chlorophenyl)*-*2*-*methyl*-*5*-*nitroso*-*1H*-*indol*-*4*-*amine* (**3b**, C_22_H_18_ClN_3_O)

Dark red unstable semisolid; MS (EI, 70 eV): *m*/*z* = 375 (M^+^, 42), 361 (55), 358 (38), 344 (12), 323 (33), 267 (9), 253 (32), 235 (36), 219 (19), 91 (100); HRMS (ESI): calcd. for C_22_H_18_ClN_3_NaO 398.1031, found 398.1040.

#### *2*-*Methyl*-*N*-*(4*-*methylphenyl)*-*5*-*nitroso*-*1*-*octyl*-*1H*-*indol*-*4*-*amine* (**3c**, C_24_H_31_N_3_O)

Dark red oil; *R*_f_ = 0.32 (toluene/ethyl acetate 10:1); ^1^H NMR (500 MHz, CDCl_3_): *δ* = 0.88 (t, *J* = 7.2 Hz, 3H), 1.26–1.32 (m, 10H), 1.70 (m, 2H), 2.16 (s, 3H), 2.42 (s, 3H), 3.93 (t, *J* = 7.7 Hz, 2H), 6.60 (d, *J* = 8.4 Hz, 2H), 6.96 (d, *J* = 8.4 Hz, 2H), 7.13–7.28 (m, 3H), 14.69 (br s, 1H) ppm; ^13^C NMR (125 MHz, CDCl_3_): *δ* = 12.43, 14.05, 22.59, 26.90, 29.12, 29.23, 30.47, 31.73, 31.78, 43.68, 104.56, 116.11, 111.92, 126.29, 129.24, 132.25, 133.76, 134.36, 135.78, 137.02, 140.54, 153.45 ppm; MS (ESI): *m*/*z* = 378 (M^+^, 100); HRMS (ESI): calcd. for C_24_H_32_N_3_O 378.2545, found 378.2548.

#### *N*-*(4*-*Chlorophenyl)*-*2*-*methyl*-*5*-*nitroso*-*1*-*octyl*-*1H*-*indol*-*4*-*amine* (**3d**, C_23_H_28_ClN_3_O)

Black solid; m.p.: 102–103 °C; *R*_f_ = 0.40 (toluene/ethyl acetate 10:1); ^1^H NMR (500 MHz, CDCl_3_): *δ* = 0.88 (t, *J* = 7.1 Hz, 3H), 1.27–1.33 (m, 10H), 1.70–1.73 (m, 2H), 2.20 (s, 3H), 3.96 (t, *J* = 7.5 Hz, 2H), 5.40 (br s, 1H), 6.91 (br s, 1H), 7.24–7.29 m, 2H), 7.34–7.42 (m, 2H), 8.13 (br s, 1H), 14.54 (s, 1H) ppm; ^13^C NMR (125 MHz, CDCl_3_): *δ* = 12.44, 14.02, 22.55, 26.86, 29.09, 29.19, 30.43, 31.70, 43.76, 104.95, 105.90, 121.14, 127.41, 128.91, 129.38, 132.29, 133.12, 134.46, 137.34, 140.80, 153.35 ppm; MS (ESI, MeOH): *m*/*z* = 398 ([M + H]^+^, 100), 380 (10); HRMS (ESI): calcd. for C_23_H_29_^35^ClN_3_O 398.1999, found 398.1997.

#### *N*-*(4*-*Methoxyphenyl)*-*2*-*methyl*-*5*-*nitroso*-*1*-*octyl*-*1H*-*indol*-*4*-*amine* (**3e**, C_24_H_31_N_3_O_2_)

Black solid; m.p.: 77–79 °C; *R*_f_ = 0.24 (toluene/ethyl acetate 10:1); ^1^H NMR (500 MHz, CDCl_3_): *δ* = 0.88 (t, *J* = 7.1 Hz, 3H), 1.26–1.32 (m, 10H), 1.67–1.71 (m, 2H), 2.16 (s, 3H), 3.87 (s, 3H), 3.94 (t, *J* = 7.8, 2H), 5.27 (s, 1H), 6.85 (d, *J* = 9.2 Hz, 1H), 6.94 (d, *J* = 8.7 Hz, 2H), 7.20 (d, *J* = 8.7 Hz, 2H), 8.06 (d, *J* = 9.2 Hz, 1H), 14.63 (s, 1H) ppm; ^13^C NMR (125 MHz, CDCl_3_): *δ* = 12.39, 14.03, 22.56, 26.87, 29.10, 29.20, 30.44, 31.71, 43.67, 55.50, 104.53, 106.00, 111.83, 114.42, 127.81, 131.10, 132.15, 133.77, 135.00, 140.50, 153.28, 158.66 ppm; MS (ESI, MeOH): *m*/*z* = 394 (M^+^, 100); HRMS (EI): calcd. for C_24_H_32_N_3_O_2_ 394.2495, found 394.2494.

#### *1*-*Benzyl*-*N*-*(5*-*methoxy*-*2*-*nitrosophenyl)*-*2*-*methyl*-*1H*-*indol*-*4*-*amine* (**7a**, C_23_H_21_N_3_O_2_)

Dark red crystals; m.p.: >115 °C (decomp); *R*_f_ = 0.48 (toluene/ethyl acetate 10:1); ^1^H NMR (500 MHz, DMSO-*d*_6_): *δ* = 2.38 (s, 3H), 3.75 (s, 3H), 5.46 (s, 2H), 6.23 (s, 1H), 6.43 (br s, 1H), 6.68 (br s, 1H), 6.90–7.04 (m, 2H), 7.12 (dd, *J* = 8.0, 7.6 Hz, 1H), 7.18 (d, *J* = 7.6 Hz, 1H), 7.21–7.25 (m, 1H), 7.27–7.32 (m, 2H), 7.36 (d, *J* = 8.0 Hz, 1H), 8.53 (br s, 1H), 13.21 (br s, 1H) ppm; ^13^C NMR (125 MHz, DMSO-*d*_6_): *δ* = 46.48, 56.43, 60.20, 95.30, 98.02, 108.86, 109.98, 115.01, 121.44, 123.99, 126.57, 127.62, 129.11, 138.40, 138.55 ppm (*spectrum not fully legible*); MS (ESI, MeOH): *m*/*z* = 394 ([M + Na]^+^), 372 ([M + H]^+^); HRMS (ESI, [M + 1]^+^): calcd. for C_23_H_22_N_3_O_2_ 372.1707, found 372.1718.

#### *1*-*Benzyl*-*N*-*(5*-*methoxy*-*2*-*nitrosophenyl)*-*2*-*methyl*-*1H*-*indol*-*5*-*amine* (**7b**, C_23_H_21_N_3_O_2_)

Dark brown crystals; yield 40 %; m.p.: >90 °C (decomp); *R*_f_ = 0.38 (toluene/ethyl acetate 10:1); ^1^H NMR (500 MHz, DMSO-*d*_6_): *δ* = 2.37 (s, 3H), 3.73 (s, 3H), 5.44 (s, 2H), 6.34 (s, 1H), 6.40 (br s, 1H), 6.64 (br s, 1H), 7.00–7.10 (m, 3H), 7.21–7.27 (m, 1H), 7.28–7.35 (m, 2H), 7.45 (d, *J* = 8.6 Hz, 1H), 7.50 (br s, 1H), 12.98 (br s, 1H) ppm; ^13^C NMR (125 MHz, DMSO-*d*_6_): *δ* = 12.49, 45.87, 55.88, 93.82, 100.39, 109.09, 110.51, 115.62, 117.91, 122.85, 125.85, 126.17, 127.13, 128.19, 128.63, 135.25, 138.17, 138.46, 142.05, 153.48, 166.67 ppm; MS (ESI, MeOH): *m*/*z* = 394 ([M + Na]^+^), 372 ([M + H]^+^); HRMS (ESI, [M + 1]^+^): calcd. for C_23_H_22_N_3_O_2_ 372.1707, found 372.1713.

### *Typical procedure for synthesis of compounds****4***

#### * 8*-*Chloro*-*2,3*-*dimethylpyrrolo[3,2*-*a]phenazine* (**4a**, C_16_H_12_ClN_3_)

To 200 mg 4-arylamino-5-nitrosoindole **3** (0.66 mmol) dissolved in 10 cm^3^ DMF was added 0.67 g *N*,*O*-bis(trimethylsilyl)acetamide (3.3 mmol). The reaction mixture was stirred at 80 °C for 12–24 h (TLC control, *n*-hexane/ethyl acetate 4:1). Then the reaction mixture was poured into 100 cm^3^ water. The product was separated, dissolved in 50 cm^3^ EtOAc, and dried with Na_2_SO_4_. After evaporation of the solvent the product was purified by column chromatography (SiO_2_, *n*-hexane/ethyl acetate 4:1). Product **4a** was obtained in the form of orange crystals; m.p.: >300 °C; *R*_f_ = 0.22 (*n*-hexane/ethyl acetate 4:1); ^1^H NMR (500 MHz, DMF-*d*_7_): *δ* = 2.59 (s, 3H), 3.96 (s, 3H), 7.18 (s, 1H), 7.76 (d, *J* = 9.4 Hz, 1H), 7.85 (dd, *J* = 9.0, 2.25 Hz, 1H), 8.18 (d, *J* = 9.4 Hz, 1H), 8.24–8.26 (m, 2H) ppm; ^13^C NMR (125 MHz, DMF-*d*_7_): *δ* = 12.33, 27.56, 102.93, 120.37, 121.59, 122.54, 128.28, 130.63, 131.22, 133.70, 135.48, 137.46, 140.45, 140.98, 141.86, 143.19 ppm; MS (EI, 70 eV): *m*/*z* = 281 (M^+^, 100), 266 (8); HRMS (EI): calcd. for C_16_H_12_ClN_3_ 281.0720, found 281.0717.

#### *3*-*Benzyl*-*8*-*chloro*-*2*-*methylpyrrolo[3,2*-*a]phenazine* (**4b**, C_22_H_16_ClN_3_)

Yellow crystals; m.p.: 223–225 °C; *R*_f_ = 0.37 (*n*-hexane/ethyl acetate 4:1); ^1^H NMR (500 MHz, CDCl_3_): *δ* = 2.50 (d, *J* = 0.8 Hz, 3H), 5.48 (s, 2H), 6.9–7.00 (m, 2H), 7.26–7.32 (m, 3H), 7.73 (dd, *J* = 9.1, 2.3 Hz, 1H), 7.77 (d, *J* = 9.3 Hz, 1H), 7.79 (d, *J* = 9.3 Hz, 1H), 8.23 (d, *J* = 2.3 Hz, 1H), 8.26 (d, *J* = 9.2 Hz, 1H) ppm; ^13^C NMR (125 MHz, CDCl_3_): *δ* = 12.86, 42.14, 103.48, 118.98, 122.00, 122.26, 125.83, 127.74, 127.87, 129.02, 130.22, 130.58, 134.09, 134.58, 136.09, 136.91, 139.71, 140.39, 141.38, 142.48 ppm; MS (ESI): *m*/*z* = 358 ([M + H]^+^); HRMS (ESI): calcd. for C_22_H_17_ClN_3_ 358.1111, found 358.1113.

#### *2,8*-*Dimethyl*-*3*-*octylpyrrolo[3,2*-*a]phenazine* (**4c**, C_24_H_29_N_3_)

Brown–red solid; m.p.: 133–135 °C; *R*_f_ = 0.54 (*n*-hexane/ethyl acetate 4:1); ^1^H NMR (500 MHz, CDCl_3_): *δ* = 0.87 (br s, 3H), 1.15–1.45 (m, 10H), 1.82 (m, 2H), 2.54 (s, 3H), 2.64 (s, 3H), 4.18 (m, 2H), 7.26 (s, 1H), 7.63 (br d, *J* = 8.0 Hz, 1H), 7.75–7.87 (m, 2H), 7.95–8.07 (m, 1H), 8.21 (br d, *J* = 8.0 Hz, 1H) ppm; ^13^C NMR (125 MHz, CDCl_3_): *δ* = 12.84, 14.01, 21.99, 22.56, 26.96, 29.13, 29.26, 30.97, 31.71, 43.88, 102.79, 118.16, 121.41, 122.00, 127.54, 128.38, 132.19, 133.81, 135.17, 138.76, 139.23, 140.52, 141.27, 141.77 ppm; MS (EI, 70 eV): *m*/*z* = 359 (M^+^, 100), 344 (7), 316 (5), 288 (8), 274 (6), 260 (47), 246 (27), 233 (99); HRMS (EI): calcd. for C_24_H_29_N_3_ 359.2361, found 359.2357.

#### *8*-*Chloro*-*2*-*methyl*-*3*-*octylpyrrolo[3,2*-*a]phenazine* (**4d**, C_23_H_26_ClN_3_)

Yellow crystals; m.p.: 157–159 °C; *R*_f_ = 0.70 (*n*-hexane/ethyl acetate 4:1); ^1^H NMR (500 MHz, CDCl_3_): *δ* = 0.86 (t, *J* = 7.1 Hz, 3H), 1.26–1.40 (m, 10H), 1.79–1.85 (m, 2H), 2.55 (s, 3H), 4.19 (t, *J* = 7.6 Hz, 2H), 7.25 (s, 1H), 7.71 (dd, *J* = 9.1, 2.2 Hz, 1H), 7.78 (d, *J* = 9.3 Hz, 1H), 7.84 (d, *J* = 9.3 Hz, 1H), 8.23–8.25 (m, 2H) ppm; ^13^C NMR (125 MHz, CDCl_3_): *δ* = 12.84, 14.01, 22.56, 26.95, 29.12, 29.25, 30.98, 31.71, 43.95, 103.11, 119.05, 121.41, 121.88, 127.79, 130.10, 130.46, 133.94, 134.06, 135.54, 139.66, 140.16, 141.20, 142.39 ppm; MS (EI, 70 eV): *m*/*z* = 379 (M^+^, 100), 282 (19), 281 (15), 266 (23); HRMS (EI): calcd. for C_23_H_26_^35^ClN_3_ 379.1815, found 379.1818.

#### *8*-*Methoxy*-*2*-*methyl*-*3*-*octylpyrrolo[3,2*-*a]phenazine* (**4e**, C_24_H_29_N_3_O)

Yellow crystals; m.p.: 122–124 °C; *R*_f_ = 0.38 (*n*-hexane/ethyl acetate 4:1); ^1^H NMR (500 MHz, CDCl_3_): *δ* = 0.86 (t, *J* = 7.1 Hz, 3H), 1.25–1.39 (m, 10H), 1.81 (m, 2H), 2.56 (s, 3H), 4.01 (s, 3H), 4.19 (t, *J* = 7.6 Hz, 2H), 7.22 (s, 1H), 7.46–7.47 (m, 2H), 7.77 (d, *J* = 9.2 Hz, 1H), 7.81 (d, *J* = 9.2 Hz, 1H), 8.16 (m, 1H) ppm; ^13^C NMR (125 MHz, CDCl_3_): *δ* = 12.86, 14.02, 22.56, 26.96, 29.13, 29.27, 30.95, 31.71, 43.83, 55.70, 102.25, 105.11, 117.89, 120.99, 122.43, 124.19, 130.07, 133.44, 135.15, 138.29, 138.89, 141.66, 142.74, 159.68 ppm; MS (EI, 70 eV): *m*/*z* = 375 (M^+^, 100), 276 (21), 262 (12), 233 (20), 219 (10); HRMS (EI): calcd. for C_24_H_29_N_3_O 375.2311, found 375.2325.

#### *2*-*Methyl*-*3*-*octyl*-*8*-*(trifluoromethyl)pyrrolo[3,2*-*a]phenazine* (**4f**, C_24_H_26_F_3_N_3_)

Orange crystals; m.p.: 127–129 °C; *R*_f_ = 0.74 (*n*-hexane/ethyl acetate 4:1); ^1^H NMR (500 MHz, CDCl_3_): *δ* = 0.87 (t, *J* = 7.1 Hz, 3H), 1.26–1.40 (m, 10H), 1.84 (m, 2H), 2.57 (s, 3H), 4.22 (t, *J* = 7.6 Hz, 2H), 7.29 (s, 1H), 7.82 (d, *J* = 9.3 Hz, 1H), 7.88 (d, *J* = 9.3 Hz, 1H), 7.93 (dd, *J* = 9.0, 2.0 Hz, 1H), 8.41 (d, *J* = 9.0 Hz, 1H), 8.58 (s, 1H) ppm; ^13^C NMR (125 MHz, CDCl_3_): *δ* = 12.87, 14.03, 22.57, 26.96, 29.13, 29.26, 31.02, 31.72, 44.03, 103.46, 119.40, 121.70, 121.75, 124.02 (q, *J* = 272 Hz), 124.49, 127.67 (q, *J* = 4.9 Hz), 129.61 (q, *J* = 32 Hz), 130.18, 134.47, 135.72, 139.79, 140.77, 142.42, 143.05 ppm; MS (EI, 70 eV): *m*/*z* = 413 (M^+^, 100), 315 (45), 301 (11), 300 (23), 287 (9); HRMS (EI): calcd. for C_24_H_26_F_3_N_3_ 413.2079, found 413.2090.

#### *3*-*Benzyl*-*9*-*methoxy*-*2*-*methylpyrrolo[3,2*-*a]phenazine* (**4g**, C_23_H_19_N_3_O)

Yield 90 %; orange crystals; m.p.: >250 °C; *R*_f_ = 0.18 (*n*-hexane/ethyl acetate 4:1); ^1^H NMR (500 MHz, DMSO-*d*_6_): *δ* = 2.48 (s, 3H), 4.03 (s, 3H), 5.65 (s, 2H), 7.04–7.08 (m, 2H), 7.18 (s, 1H), 7.23–7.35 (m, 3H), 7.52 (dd, *J* = 9.3, 2.5 Hz, 1H), 7.57 (d, *J* = 2.5 Hz, 1H), 7.72 (d, *J* = 9.0 Hz, 1H), 8.08 (d, *J* = 9.3 Hz, 1H), 8.10 (d, *J* = 9.0 Hz, 1H) ppm; ^13^C NMR (125 MHz, DMSO-*d*_6_): *δ* = 13.05, 46.82, 56.37, 102.99, 105.45, 118.18, 121.75, 121.94, 123.76, 126.66, 127.77, 129.22, 130.80, 135.02, 136.36, 138.05, 138.43, 139.43, 140.03, 143.53, 160.72 ppm; MS (ESI): *m*/*z* = 354 ([M + H]^+^); HRMS (ESI): calcd. for C_23_H_20_N_3_O 354.1601, found 354.1615.

#### *3*-*Benzyl*-*8*-*methoxy*-*2*-*methylpyrrolo[3,2*-*a]phenazine* (**4h**, C_23_H_19_N_3_O)

Yield 64 %; yellow crystals; m.p.: 225–227 °C; *R*_f_ = 0.22 (*n*-hexane/ethyl acetate 4:1); ^1^H NMR (500 MHz, CDCl_3_): *δ* = 2.48 (d, *J* = 0.7 Hz, 3H), 4.01 (s, 3H), 5.45 (s, 2H), 6.97 (br s, 1H), 7.22–7.30 (m, 3H), 7.32 (s, 1H), 7.45–7.50 (m, 2H), 7.73 (d, *J* = 9.3 Hz, 1H), 7.75 (d, *J* = 9.3 Hz, 1H), 8.18 (d, *J* = 9.0 Hz, 1H) ppm; ^13^C NMR (125 MHz, CDCl_3_): *δ* = 12.83, 47.00, 55.73, 102.75, 105.06, 117.86, 121.47, 122.73, 124.38, 125.83, 127.59, 128.93, 130.09, 133.98, 135.72, 137.17, 138.20, 138.98, 141.65, 142.82, 159.08 ppm; MS (ESI): *m*/*z* = 354 ([M + H]^+^); HRMS (ESI): calcd. for C_23_H_20_N_3_O 354.1601, found 354.1604.
